# Crystal structure and absolute configuration of preaustinoid A1

**DOI:** 10.1107/S2056989015013614

**Published:** 2015-07-22

**Authors:** Andrea Stierle, Donald Stierle, Daniel Decato

**Affiliations:** a Department of Biological and Pharmaceutical Sciences, University of Montana, 32 Campus Drive, Missoula, Montana 59812, USA; b Department of Chemistry and Biochemistry, University of Montana, 32 Campus Drive, Missoula, Montana 59812, USA

**Keywords:** crystal structure, meroterpene, preaustinoid A1, absolute configuration, hydrogen bonding, helical chain

## Abstract

The absolute structure of the title compound preaustinoid A1 [systematic name: (5a*R*,7a*S*,8*R*,10*S*,12*R*,13a*R*,13b*S*)-methyl 10-hy­droxy-5,5,7a,10,12,13b-hexa­methyl-14-methyl­ene-3,9,11-trioxohexa­deca­hydro-8,12-methano­cyclo­octa­[3,4]benzo[1,2-*c*]oxepine-8-carboxyl­ate], C_26_H_36_O_7_, has been determined by resonant scattering using Cu *K*α radiation [Flack parameter = 0.07 (15)]. The structure is consistent with that reported previously [Stierle *et al.* (2011). *J. Nat. Prod.* **74**, 2272–2277], determined by detailed analysis of MS and NMR data. The mol­ecule consists of a fused four-ring arrangement. The seven-membered oxepan-2-one ring has a chair conformation, as do the central cyclo­hexane rings, while the outer cyclo­hexa-1,3-dione ring has a boat conformation. In the crystal, mol­ecules are linked *via* O—H⋯O hydrogen bonds, forming helical chains propagating along [100].

## Related literature   

For the structure of the title compound determined by detailed analysis of MS and NMR data, see: Stierle *et al.* (2011 ▸). For other details concerning preaustinoid A1, see: Geris dos Santos *et al.* (2003 ▸). For the crystal structure of the closely related compound preaustinoid A, for which the absolute configuration was assigned based solely on the optical rotation of the mol­ecule, see: Maganhi *et al.* (2009 ▸). For the characterization of preaustinoid A, see: Geris dos Santos *et al.* (2002 ▸); Stierle *et al.* (2011 ▸). For the absolute configuration of a closely related meroterpene, berkeleydione, based on the helicity rule of circular dichroism, see: Stierle *et al.* (2011 ▸). For details of its characterization, see: Stierle *et al.* (2004 ▸), and for its crystal structure and absolute configuration determined by resonant scattering, see: Stierle *et al.* (2015 ▸). The absolute configuration reported here is consistent with that of related meroterpenes including berkeleydione (Stierle *et al.*, 2015 ▸), dhirolide A (de Silva *et al.*, 2011 ▸) and minuteolide A (Iida *et al.*, 2008 ▸).
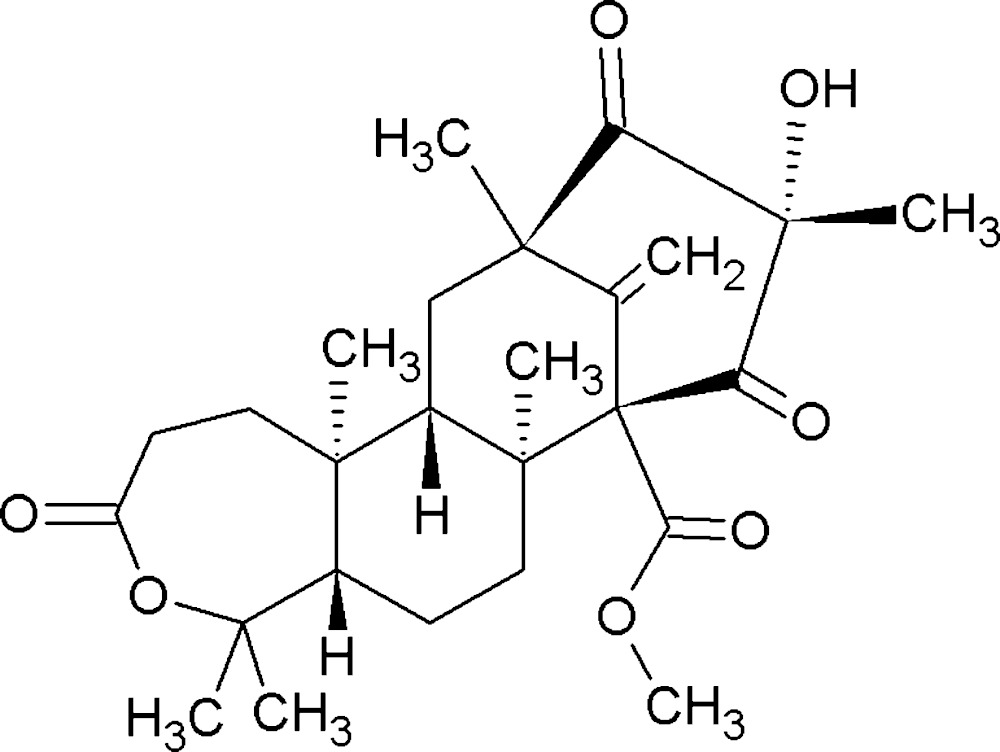


## Experimental   

### Crystal data  

C_26_H_36_O_7_
*M* *_r_* = 460.55
Orthorhombic, 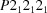

*a* = 8.3169 (4) Å
*b* = 13.8064 (6) Å
*c* = 19.9243 (9) Å
*V* = 2287.84 (18) Å^3^
*Z* = 4
Cu *K*α radiation
μ = 0.79 mm^−1^
*T* = 100 K
0.25 × 0.25 × 0.05 mm

### Data collection  

Bruker D8 Venture diffractometer
Absorption correction: multi-scan (*SADABS*; Bruker, 2012 ▸) *T*_min_ = 0.644, *T*_max_ = 0.753
28885 measured reflections
4008 independent reflections
3740 reflections with *I* > 2σ(*I*)
*R*_int_ = 0.069

### Refinement  

*R*[*F* ^2^ > 2σ(*F* ^2^)] = 0.057
*wR*(*F* ^2^) = 0.120
*S* = 1.17
4008 reflections
309 parameters
H atoms treated by a mixture of independent and constrained refinement
Δρ_max_ = 0.51 e Å^−3^
Δρ_min_ = −0.21 e Å^−3^
Absolute structure: Flack *x* determined using 1409 quotients [(*I* ^+^)−(*I* ^−^)]/[(*I* ^+^)+(*I* ^−^)] (Parsons *et al.*, 2013 ▸)
Absolute structure parameter: 0.07 (15)

### 

Data collection: *APEX2* (Bruker, 2012 ▸); cell refinement: *SAINT* (Bruker, 2012 ▸); data reduction: *SAINT*; program(s) used to solve structure: *SHELXT* (Sheldrick, 2015*a* ▸); program(s) used to refine structure: *SHELXL2014* (Sheldrick, 2015*b* ▸); molecular graphics: *OLEX2* (Dolomanov *et al.*, 2009 ▸); software used to prepare material for publication: *OLEX2*.

## Supplementary Material

Crystal structure: contains datablock(s) Global, I. DOI: 10.1107/S2056989015013614/su5167sup1.cif

Structure factors: contains datablock(s) I. DOI: 10.1107/S2056989015013614/su5167Isup2.hkl

**Fig. 1.**
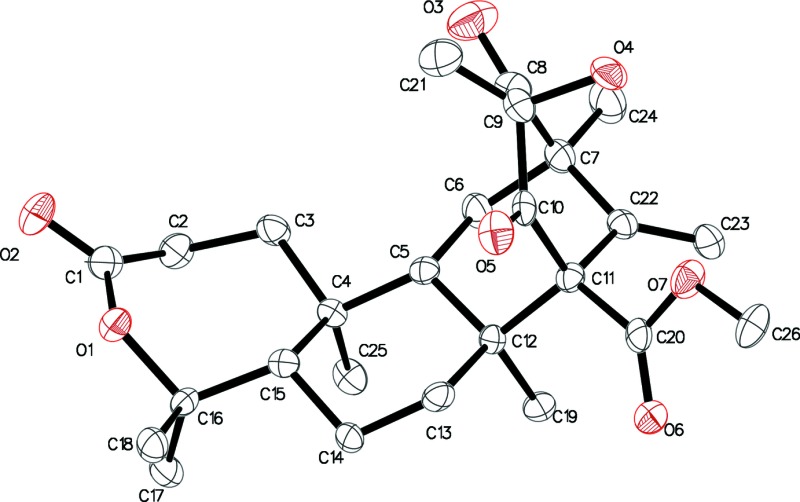
Molecular structure of the title compound, with atom labelling. Displacement ellipsoids are drawn at the 50% probability level. Hydrogen atoms have been omitted for clarity.

. DOI: 10.1107/S2056989015013614/su5167fig1.tif
Mol­ecular structure of the title compound, with atom labelling. Displacement ellipsoids are drawn at the 50% probability level. Hydrogen atoms have been omitted for clarity.

CCDC reference: 1405963

Additional supporting information:  crystallographic information; 3D view; checkCIF report

## Figures and Tables

**Table 1:** Hydrogen-bond geometry (, )

*D*H*A*	*D*H	H*A*	*D* *A*	*D*H*A*
O4H4O2^i^	0.84(7)	1.89(7)	2.723(5)	168(6)

Symmetry code: (i) 
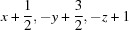
.

## supplementary crystallographic information

### S1. Synthesis and crystallization

Berkeleydione, preaustinoid A and the title compound, preaustinoid A1, were co-isolated from the organic extract of *Penicillium rubrum* (Stierle *et al.* 2011). Colorless crystals of the title compound were grown by vapor diffusion of pentane into a chloro­form solution.

### S2. Refinement

All the H atoms were located in difference Fourier maps and the hydroxyl H atom was freely refined. The C-bound H atoms were finally placed in calculated positions and refined using a riding model: C—H = 0.95 - 1.0 Å with U_iso_(H) = 1.5U_eq_(C) for methyl H atoms and 1.2U_eq_(C) for other H atoms.

### S3. Comment

The absolute configuration of the title compound preasutinoid A1, has been determined by X-ray by refinement of the Flack parameter with data collected using Cu Kα radiation. The absolute configuration reported here is consistent with that of related meroterpenes including berkeleydione (Stierle *et al.*, 2015), dhirolide A (de Silva *et al.*, 2011) and minuteolide A (Iida *et al.*, 2008).

The title molecule, Fig. 1, consists of a fused four-ring arrangement. The seven-membered oxepan-2-one ring (O1/C1—C4/C15/C16) has a chair conformation, as do the central cyclo­hexane rings (C4/C5/C12/C15 and C5—C7/C22/C11/C12), while the outer cyclo­hexa-1,3-dione ring (C7—C11/C22) has a boat conformation.

In the crystal, molecules are linked via O—H···O hydrogen bonds forming helices propagating along [100]; see Table 1.

### Crystal data

**Table:**

C_26_H_36_O_7_	*D*_x_ = 1.337 Mg m^−^^3^
*M**_r_* = 460.55	Cu *K*α radiation, λ = 1.54178 Å
Orthorhombic, *P*2_1_2_1_2_1_	Cell parameters from 9895 reflections
*a* = 8.3169 (4) Å	θ = 3.9–66.6°
*b* = 13.8064 (6) Å	µ = 0.79 mm^−^^1^
*c* = 19.9243 (9) Å	*T* = 100 K
*V* = 2287.84 (18) Å^3^	Plate, colorless
*Z* = 4	0.25 × 0.25 × 0.05 mm
*F*(000) = 992	

### Data collection

**Table:**

Bruker D8 Venture diffractometer	4008 independent reflections
Radiation source: microfocus sealed X-ray tube, Incoatec Iµus	3740 reflections with *I* > 2σ(*I*)
Double Bounce Multilayer Mirror monochromator	*R*_int_ = 0.069
Detector resolution: 10.5 pixels mm^-1^	θ_max_ = 66.6°, θ_min_ = 3.9°
ω and φ scans	*h* = −9→9
Absorption correction: multi-scan (*SADABS*; Bruker, 2012)	*k* = −16→16
*T*_min_ = 0.644, *T*_max_ = 0.753	*l* = −22→23
28885 measured reflections	

### Refinement

**Table:**

Refinement on *F*^2^	Hydrogen site location: mixed
Least-squares matrix: full	H atoms treated by a mixture of independent and constrained refinement
*R*[*F*^2^ > 2σ(*F*^2^)] = 0.057	*w* = 1/[σ^2^(*F*_o_^2^) + (0.0318*P*)^2^ + 2.3628*P*] where *P* = (*F*_o_^2^ + 2*F*_c_^2^)/3
*wR*(*F*^2^) = 0.120	(Δ/σ)_max_ < 0.001
*S* = 1.17	Δρ_max_ = 0.51 e Å^−^^3^
4008 reflections	Δρ_min_ = −0.21 e Å^−^^3^
309 parameters	Absolute structure: Flack *x* determined using 1409 quotients [(*I*^+^)-(*I*^-^)]/[(*I*^+^)+(*I*^-^)] (Parsons *et al.*, 2013)
0 restraints	Absolute structure parameter: 0.07 (15)
Primary atom site location: structure-invariant direct methods	

### Special details

**Table:**

Geometry. All e.s.d.'s (except the e.s.d. in the dihedral angle between two l.s. planes) are estimated using the full covariance matrix. The cell e.s.d.'s are taken into account individually in the estimation of e.s.d.'s in distances, angles and torsion angles; correlations between e.s.d.'s in cell parameters are only used when they are defined by crystal symmetry. An approximate (isotropic) treatment of cell e.s.d.'s is used for estimating e.s.d.'s involving l.s. planes.

### Fractional atomic coordinates and isotropic or equivalent isotropic displacement parameters (Å^2^)

**Table:**

	*x*	*y*	*z*	*U*_iso_*/*U*_eq_	
O1	0.2993 (3)	0.6369 (2)	0.29734 (14)	0.0240 (6)	
O2	0.3728 (4)	0.7808 (2)	0.26743 (16)	0.0341 (8)	
O3	0.7964 (5)	0.7142 (3)	0.56157 (18)	0.0472 (10)	
O4	0.6672 (4)	0.5863 (2)	0.67932 (16)	0.0357 (8)	
O5	0.3884 (3)	0.5028 (2)	0.60306 (15)	0.0296 (7)	
O6	0.5590 (4)	0.2577 (2)	0.58385 (15)	0.0299 (7)	
O7	0.5763 (4)	0.3582 (2)	0.67092 (14)	0.0293 (7)	
C1	0.4147 (5)	0.7002 (3)	0.2841 (2)	0.0255 (10)	
C2	0.5872 (5)	0.6749 (3)	0.2919 (2)	0.0267 (10)	
H2A	0.6530	0.7327	0.2815	0.032*	
H2B	0.6150	0.6240	0.2589	0.032*	
C3	0.6311 (5)	0.6389 (3)	0.3623 (2)	0.0240 (9)	
H3A	0.5729	0.6795	0.3953	0.029*	
H3B	0.7474	0.6504	0.3692	0.029*	
C4	0.5961 (5)	0.5318 (3)	0.37889 (19)	0.0184 (8)	
C5	0.6433 (5)	0.5178 (3)	0.4548 (2)	0.0181 (8)	
H5	0.5770	0.5659	0.4801	0.022*	
C6	0.8199 (5)	0.5452 (3)	0.4698 (2)	0.0255 (9)	
H6A	0.8423	0.6099	0.4504	0.031*	
H6B	0.8918	0.4980	0.4474	0.031*	
C7	0.8590 (5)	0.5472 (3)	0.5463 (2)	0.0266 (10)	
C8	0.7589 (6)	0.6306 (3)	0.5724 (2)	0.0322 (11)	
C9	0.6118 (6)	0.6076 (3)	0.6130 (2)	0.0312 (11)	
C10	0.5297 (5)	0.5122 (3)	0.5936 (2)	0.0201 (9)	
C11	0.6330 (5)	0.4282 (3)	0.5655 (2)	0.0188 (9)	
C12	0.6024 (5)	0.4176 (3)	0.48660 (19)	0.0177 (8)	
C13	0.4256 (5)	0.3930 (3)	0.4717 (2)	0.0217 (9)	
H13A	0.4050	0.3249	0.4847	0.026*	
H13B	0.3557	0.4347	0.4997	0.026*	
C14	0.3804 (5)	0.4064 (3)	0.3988 (2)	0.0215 (9)	
H14A	0.2652	0.3905	0.3929	0.026*	
H14B	0.4436	0.3607	0.3710	0.026*	
C15	0.4109 (5)	0.5100 (3)	0.3743 (2)	0.0186 (8)	
H15	0.3599	0.5527	0.4088	0.022*	
C16	0.3177 (5)	0.5300 (3)	0.3086 (2)	0.0206 (9)	
C17	0.3772 (5)	0.4821 (3)	0.2451 (2)	0.0263 (10)	
H17A	0.3031	0.4965	0.2082	0.039*	
H17B	0.3828	0.4119	0.2519	0.039*	
H17C	0.4845	0.5068	0.2341	0.039*	
C18	0.1390 (5)	0.5056 (3)	0.3176 (2)	0.0264 (10)	
H18A	0.1007	0.5325	0.3602	0.040*	
H18B	0.1250	0.4351	0.3178	0.040*	
H18C	0.0772	0.5336	0.2805	0.040*	
C19	0.7085 (5)	0.3349 (3)	0.4592 (2)	0.0239 (9)	
H19A	0.6839	0.3243	0.4117	0.036*	
H19B	0.6870	0.2754	0.4845	0.036*	
H19C	0.8221	0.3525	0.4640	0.036*	
C20	0.5829 (5)	0.3371 (3)	0.6056 (2)	0.0217 (9)	
C21	0.4904 (7)	0.6909 (4)	0.6118 (3)	0.0409 (13)	
H21A	0.4027	0.6769	0.6432	0.061*	
H21B	0.4466	0.6979	0.5663	0.061*	
H21C	0.5439	0.7511	0.6250	0.061*	
C22	0.8113 (5)	0.4504 (3)	0.5764 (2)	0.0198 (9)	
C23	0.9147 (5)	0.3889 (3)	0.6029 (2)	0.0262 (10)	
H23A	1.0261	0.4043	0.6039	0.031*	
H23B	0.8779	0.3293	0.6210	0.031*	
C24	1.0372 (6)	0.5727 (4)	0.5545 (3)	0.0407 (13)	
H24A	1.1033	0.5210	0.5352	0.061*	
H24B	1.0623	0.5797	0.6023	0.061*	
H24C	1.0599	0.6337	0.5312	0.061*	
C25	0.7018 (5)	0.4690 (3)	0.3313 (2)	0.0247 (9)	
H25A	0.8081	0.4595	0.3515	0.037*	
H25B	0.7136	0.5019	0.2880	0.037*	
H25C	0.6503	0.4059	0.3244	0.037*	
C26	0.5265 (6)	0.2787 (3)	0.7134 (2)	0.0325 (11)	
H26A	0.5114	0.3021	0.7594	0.049*	
H26B	0.6092	0.2282	0.7129	0.049*	
H26C	0.4250	0.2519	0.6966	0.049*	
H4	0.726 (7)	0.633 (5)	0.692 (3)	0.055 (18)*	

### Atomic displacement parameters (Å^2^)

**Table:**

	*U*^11^	*U*^22^	*U*^33^	*U*^12^	*U*^13^	*U*^23^
O1	0.0242 (15)	0.0203 (14)	0.0276 (16)	0.0008 (12)	0.0005 (12)	0.0030 (12)
O2	0.0386 (19)	0.0246 (17)	0.0390 (19)	0.0014 (14)	0.0037 (15)	0.0119 (14)
O3	0.063 (2)	0.0293 (19)	0.049 (2)	−0.0072 (18)	0.0082 (19)	0.0000 (16)
O4	0.057 (2)	0.0309 (17)	0.0195 (17)	−0.0106 (17)	−0.0101 (16)	0.0005 (13)
O5	0.0208 (16)	0.0391 (18)	0.0288 (17)	0.0086 (14)	0.0053 (13)	0.0015 (13)
O6	0.0353 (18)	0.0224 (16)	0.0319 (18)	−0.0034 (13)	−0.0075 (15)	0.0039 (13)
O7	0.0398 (18)	0.0251 (15)	0.0230 (16)	0.0046 (14)	0.0054 (13)	0.0046 (12)
C1	0.034 (2)	0.025 (2)	0.018 (2)	−0.0053 (19)	0.0031 (19)	0.0024 (17)
C2	0.029 (2)	0.025 (2)	0.026 (2)	−0.0058 (18)	0.003 (2)	0.0056 (17)
C3	0.024 (2)	0.025 (2)	0.023 (2)	−0.0084 (18)	−0.0030 (18)	0.0018 (17)
C4	0.0175 (19)	0.0197 (19)	0.018 (2)	−0.0014 (16)	−0.0006 (16)	−0.0012 (15)
C5	0.018 (2)	0.017 (2)	0.019 (2)	0.0016 (15)	0.0014 (16)	−0.0022 (15)
C6	0.022 (2)	0.028 (2)	0.026 (2)	−0.0040 (19)	−0.0053 (18)	0.0052 (18)
C7	0.022 (2)	0.027 (2)	0.030 (2)	−0.0021 (18)	−0.0088 (18)	0.0018 (18)
C8	0.042 (3)	0.027 (3)	0.027 (3)	−0.003 (2)	−0.007 (2)	−0.0038 (19)
C9	0.043 (3)	0.029 (2)	0.021 (2)	−0.002 (2)	−0.009 (2)	0.0005 (18)
C10	0.022 (2)	0.024 (2)	0.014 (2)	0.0053 (17)	−0.0009 (16)	0.0039 (16)
C11	0.020 (2)	0.018 (2)	0.018 (2)	0.0025 (16)	0.0010 (16)	−0.0003 (15)
C12	0.0166 (19)	0.0178 (19)	0.019 (2)	0.0021 (17)	−0.0018 (16)	0.0001 (15)
C13	0.021 (2)	0.017 (2)	0.027 (2)	−0.0040 (16)	0.0007 (17)	0.0026 (16)
C14	0.0164 (19)	0.021 (2)	0.027 (2)	−0.0040 (17)	−0.0073 (17)	0.0030 (16)
C15	0.0164 (19)	0.019 (2)	0.021 (2)	0.0000 (16)	0.0010 (16)	−0.0025 (15)
C16	0.023 (2)	0.0136 (18)	0.025 (2)	−0.0003 (16)	−0.0026 (17)	0.0027 (16)
C17	0.029 (2)	0.029 (2)	0.021 (2)	0.0005 (19)	−0.0030 (18)	−0.0029 (17)
C18	0.021 (2)	0.027 (2)	0.031 (2)	−0.0015 (18)	−0.0058 (18)	0.0035 (19)
C19	0.026 (2)	0.020 (2)	0.025 (2)	0.0050 (17)	−0.0016 (18)	−0.0048 (17)
C20	0.016 (2)	0.023 (2)	0.027 (2)	0.0039 (17)	−0.0050 (17)	0.0026 (17)
C21	0.051 (3)	0.031 (3)	0.040 (3)	0.006 (2)	−0.001 (2)	−0.007 (2)
C22	0.020 (2)	0.022 (2)	0.018 (2)	0.0026 (17)	−0.0007 (17)	−0.0036 (16)
C23	0.021 (2)	0.029 (2)	0.028 (2)	0.0005 (18)	−0.0060 (18)	−0.0007 (18)
C24	0.032 (3)	0.044 (3)	0.046 (3)	−0.008 (2)	−0.014 (2)	0.009 (2)
C25	0.017 (2)	0.033 (2)	0.024 (2)	0.0024 (18)	0.0009 (17)	−0.0032 (18)
C26	0.038 (3)	0.026 (2)	0.033 (3)	0.005 (2)	0.007 (2)	0.013 (2)

### Geometric parameters (Å, º)

**Table:**

O1—C1	1.324 (5)	C12—C13	1.538 (5)
O1—C16	1.501 (5)	C12—C19	1.542 (5)
O2—C1	1.213 (5)	C13—H13A	0.9900
O3—C8	1.214 (6)	C13—H13B	0.9900
O4—C9	1.431 (5)	C13—C14	1.511 (6)
O4—H4	0.84 (7)	C14—H14A	0.9900
O5—C10	1.198 (5)	C14—H14B	0.9900
O6—C20	1.196 (5)	C14—C15	1.532 (5)
O7—C20	1.335 (5)	C15—H15	1.0000
O7—C26	1.446 (5)	C15—C16	1.547 (6)
C1—C2	1.485 (6)	C16—C17	1.511 (6)
C2—H2A	0.9900	C16—C18	1.534 (6)
C2—H2B	0.9900	C17—H17A	0.9800
C2—C3	1.532 (6)	C17—H17B	0.9800
C3—H3A	0.9900	C17—H17C	0.9800
C3—H3B	0.9900	C18—H18A	0.9800
C3—C4	1.543 (5)	C18—H18B	0.9800
C4—C5	1.575 (5)	C18—H18C	0.9800
C4—C15	1.572 (5)	C19—H19A	0.9800
C4—C25	1.558 (6)	C19—H19B	0.9800
C5—H5	1.0000	C19—H19C	0.9800
C5—C6	1.546 (5)	C21—H21A	0.9800
C5—C12	1.559 (5)	C21—H21B	0.9800
C6—H6A	0.9900	C21—H21C	0.9800
C6—H6B	0.9900	C22—C23	1.319 (6)
C6—C7	1.559 (6)	C23—H23A	0.9500
C7—C8	1.513 (7)	C23—H23B	0.9500
C7—C22	1.519 (6)	C24—H24A	0.9800
C7—C24	1.532 (6)	C24—H24B	0.9800
C8—C9	1.500 (7)	C24—H24C	0.9800
C9—C10	1.534 (6)	C25—H25A	0.9800
C9—C21	1.530 (7)	C25—H25B	0.9800
C10—C11	1.547 (5)	C25—H25C	0.9800
C11—C12	1.600 (5)	C26—H26A	0.9800
C11—C20	1.547 (6)	C26—H26B	0.9800
C11—C22	1.529 (6)	C26—H26C	0.9800
			
C1—O1—C16	127.2 (3)	C14—C13—H13B	108.9
C9—O4—H4	107 (4)	C13—C14—H14A	109.2
C20—O7—C26	114.6 (3)	C13—C14—H14B	109.2
O1—C1—C2	121.6 (4)	C13—C14—C15	112.2 (3)
O2—C1—O1	116.8 (4)	H14A—C14—H14B	107.9
O2—C1—C2	121.5 (4)	C15—C14—H14A	109.2
C1—C2—H2A	108.8	C15—C14—H14B	109.2
C1—C2—H2B	108.8	C4—C15—H15	105.2
C1—C2—C3	113.8 (4)	C14—C15—C4	108.8 (3)
H2A—C2—H2B	107.7	C14—C15—H15	105.2
C3—C2—H2A	108.8	C14—C15—C16	110.7 (3)
C3—C2—H2B	108.8	C16—C15—C4	120.4 (3)
C2—C3—H3A	107.9	C16—C15—H15	105.2
C2—C3—H3B	107.9	O1—C16—C15	110.7 (3)
C2—C3—C4	117.5 (3)	O1—C16—C17	109.8 (3)
H3A—C3—H3B	107.2	O1—C16—C18	97.7 (3)
C4—C3—H3A	107.9	C17—C16—C15	117.8 (3)
C4—C3—H3B	107.9	C17—C16—C18	108.6 (4)
C3—C4—C5	106.1 (3)	C18—C16—C15	110.3 (3)
C3—C4—C15	110.9 (3)	C16—C17—H17A	109.5
C3—C4—C25	107.3 (3)	C16—C17—H17B	109.5
C15—C4—C5	106.0 (3)	C16—C17—H17C	109.5
C25—C4—C5	112.0 (3)	H17A—C17—H17B	109.5
C25—C4—C15	114.3 (3)	H17A—C17—H17C	109.5
C4—C5—H5	105.3	H17B—C17—H17C	109.5
C6—C5—C4	113.1 (3)	C16—C18—H18A	109.5
C6—C5—H5	105.3	C16—C18—H18B	109.5
C6—C5—C12	110.3 (3)	C16—C18—H18C	109.5
C12—C5—C4	116.4 (3)	H18A—C18—H18B	109.5
C12—C5—H5	105.3	H18A—C18—H18C	109.5
C5—C6—H6A	109.0	H18B—C18—H18C	109.5
C5—C6—H6B	109.0	C12—C19—H19A	109.5
C5—C6—C7	113.0 (3)	C12—C19—H19B	109.5
H6A—C6—H6B	107.8	C12—C19—H19C	109.5
C7—C6—H6A	109.0	H19A—C19—H19B	109.5
C7—C6—H6B	109.0	H19A—C19—H19C	109.5
C8—C7—C6	103.6 (4)	H19B—C19—H19C	109.5
C8—C7—C22	113.0 (4)	O6—C20—O7	123.1 (4)
C8—C7—C24	108.7 (4)	O6—C20—C11	127.1 (4)
C22—C7—C6	108.4 (3)	O7—C20—C11	109.7 (3)
C22—C7—C24	114.4 (4)	C9—C21—H21A	109.5
C24—C7—C6	108.1 (4)	C9—C21—H21B	109.5
O3—C8—C7	121.4 (5)	C9—C21—H21C	109.5
O3—C8—C9	120.4 (5)	H21A—C21—H21B	109.5
C9—C8—C7	118.2 (4)	H21A—C21—H21C	109.5
O4—C9—C8	106.2 (4)	H21B—C21—H21C	109.5
O4—C9—C10	101.5 (3)	C7—C22—C11	111.9 (3)
O4—C9—C21	112.4 (4)	C23—C22—C7	123.7 (4)
C8—C9—C10	114.2 (4)	C23—C22—C11	124.1 (4)
C8—C9—C21	111.7 (4)	C22—C23—H23A	120.0
C21—C9—C10	110.3 (4)	C22—C23—H23B	120.0
O5—C10—C9	119.4 (4)	H23A—C23—H23B	120.0
O5—C10—C11	121.4 (4)	C7—C24—H24A	109.5
C9—C10—C11	119.2 (3)	C7—C24—H24B	109.5
C10—C11—C12	109.6 (3)	C7—C24—H24C	109.5
C20—C11—C10	105.9 (3)	H24A—C24—H24B	109.5
C20—C11—C12	112.9 (3)	H24A—C24—H24C	109.5
C22—C11—C10	109.7 (3)	H24B—C24—H24C	109.5
C22—C11—C12	108.2 (3)	C4—C25—H25A	109.5
C22—C11—C20	110.5 (3)	C4—C25—H25B	109.5
C5—C12—C11	106.4 (3)	C4—C25—H25C	109.5
C13—C12—C5	109.0 (3)	H25A—C25—H25B	109.5
C13—C12—C11	111.2 (3)	H25A—C25—H25C	109.5
C13—C12—C19	108.4 (3)	H25B—C25—H25C	109.5
C19—C12—C5	112.8 (3)	O7—C26—H26A	109.5
C19—C12—C11	109.0 (3)	O7—C26—H26B	109.5
C12—C13—H13A	108.9	O7—C26—H26C	109.5
C12—C13—H13B	108.9	H26A—C26—H26B	109.5
H13A—C13—H13B	107.7	H26A—C26—H26C	109.5
C14—C13—C12	113.3 (3)	H26B—C26—H26C	109.5
C14—C13—H13A	108.9		
			
O1—C1—C2—C3	−56.1 (5)	C9—C10—C11—C22	12.1 (5)
O2—C1—C2—C3	121.3 (4)	C10—C11—C12—C5	57.0 (4)
O3—C8—C9—O4	−99.4 (5)	C10—C11—C12—C13	−61.6 (4)
O3—C8—C9—C10	149.6 (4)	C10—C11—C12—C19	179.0 (3)
O3—C8—C9—C21	23.6 (6)	C10—C11—C20—O6	137.0 (4)
O4—C9—C10—O5	93.3 (5)	C10—C11—C20—O7	−45.9 (4)
O4—C9—C10—C11	−83.5 (4)	C10—C11—C22—C7	−55.2 (4)
O5—C10—C11—C12	76.7 (5)	C10—C11—C22—C23	130.9 (4)
O5—C10—C11—C20	−45.4 (5)	C11—C12—C13—C14	165.7 (3)
O5—C10—C11—C22	−164.7 (4)	C12—C5—C6—C7	−56.9 (4)
C1—O1—C16—C15	67.1 (5)	C12—C11—C20—O6	17.0 (6)
C1—O1—C16—C17	−64.7 (5)	C12—C11—C20—O7	−165.8 (3)
C1—O1—C16—C18	−177.7 (4)	C12—C11—C22—C7	64.3 (4)
C1—C2—C3—C4	81.5 (5)	C12—C11—C22—C23	−109.6 (5)
C2—C3—C4—C5	−176.7 (3)	C12—C13—C14—C15	−57.8 (5)
C2—C3—C4—C15	−62.0 (5)	C13—C14—C15—C4	63.0 (4)
C2—C3—C4—C25	63.4 (5)	C13—C14—C15—C16	−162.6 (3)
C3—C4—C5—C6	−57.0 (4)	C14—C15—C16—O1	160.3 (3)
C3—C4—C5—C12	173.8 (3)	C14—C15—C16—C17	−72.2 (4)
C3—C4—C15—C14	−173.7 (3)	C14—C15—C16—C18	53.3 (4)
C3—C4—C15—C16	57.1 (4)	C15—C4—C5—C6	−174.9 (3)
C4—C5—C6—C7	170.8 (3)	C15—C4—C5—C12	55.8 (4)
C4—C5—C12—C11	−170.3 (3)	C16—O1—C1—O2	170.0 (4)
C4—C5—C12—C13	−50.2 (4)	C16—O1—C1—C2	−12.5 (6)
C4—C5—C12—C19	70.3 (4)	C19—C12—C13—C14	−74.5 (4)
C4—C15—C16—O1	−71.3 (4)	C20—C11—C12—C5	174.8 (3)
C4—C15—C16—C17	56.2 (5)	C20—C11—C12—C13	56.1 (4)
C4—C15—C16—C18	−178.3 (3)	C20—C11—C12—C19	−63.3 (4)
C5—C4—C15—C14	−59.0 (4)	C20—C11—C22—C7	−171.6 (3)
C5—C4—C15—C16	171.8 (3)	C20—C11—C22—C23	14.5 (6)
C5—C6—C7—C8	−66.2 (4)	C21—C9—C10—O5	−26.1 (5)
C5—C6—C7—C22	54.1 (5)	C21—C9—C10—C11	157.1 (4)
C5—C6—C7—C24	178.6 (4)	C22—C7—C8—O3	168.6 (4)
C5—C12—C13—C14	48.6 (4)	C22—C7—C8—C9	−10.7 (6)
C6—C5—C12—C11	59.2 (4)	C22—C11—C12—C5	−62.6 (4)
C6—C5—C12—C13	179.2 (3)	C22—C11—C12—C13	178.8 (3)
C6—C5—C12—C19	−60.3 (4)	C22—C11—C12—C19	59.4 (4)
C6—C7—C8—O3	−74.3 (5)	C22—C11—C20—O6	−104.3 (5)
C6—C7—C8—C9	106.4 (4)	C22—C11—C20—O7	72.9 (4)
C6—C7—C22—C11	−58.3 (4)	C24—C7—C8—O3	40.5 (6)
C6—C7—C22—C23	115.6 (5)	C24—C7—C8—C9	−138.8 (4)
C7—C8—C9—O4	79.9 (5)	C24—C7—C22—C11	−179.0 (4)
C7—C8—C9—C10	−31.1 (6)	C24—C7—C22—C23	−5.1 (6)
C7—C8—C9—C21	−157.1 (4)	C25—C4—C5—C6	59.8 (4)
C8—C7—C22—C11	55.9 (5)	C25—C4—C5—C12	−69.5 (4)
C8—C7—C22—C23	−130.2 (4)	C25—C4—C15—C14	64.9 (4)
C8—C9—C10—O5	−152.9 (4)	C25—C4—C15—C16	−64.3 (5)
C8—C9—C10—C11	30.3 (5)	C26—O7—C20—O6	−4.2 (6)
C9—C10—C11—C12	−106.5 (4)	C26—O7—C20—C11	178.5 (3)
C9—C10—C11—C20	131.4 (4)		

### Hydrogen-bond geometry (Å, º)

**Table:**

*D*—H···*A*	*D*—H	H···*A*	*D*···*A*	*D*—H···*A*
O4—H4···O2^i^	0.84 (7)	1.89 (7)	2.723 (5)	168 (6)

Symmetry code: (i) *x*+1/2, −*y*+3/2, −*z*+1.
