# Supplementary figures and images for: Crystal structure and absolute configuration of preaustinoid A1
Source: Acta Crystallogr E Crystallogr Commun. 2015 Jul 22;71(Pt 8):o596–7. doi: 10.1107/S2056989015013614 (PMC4571416; doi:10.1107/S2056989015013614)

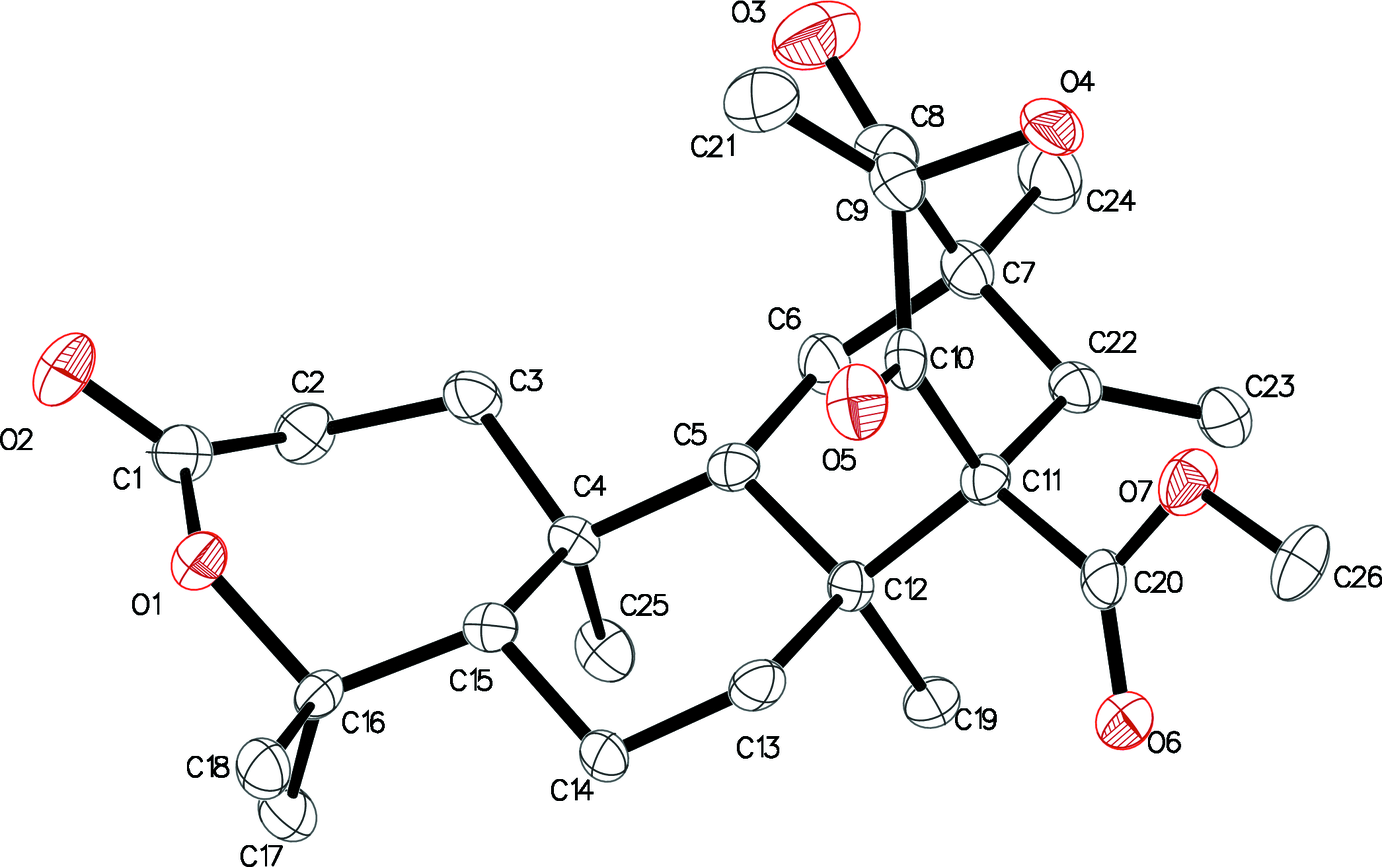

Supplement: Supplementary file 3 [file e-71-0o596-fig1.tif]
